# Intestinal microbiota composition after antibiotic treatment in early life: the INCA study

**DOI:** 10.1186/s12887-015-0519-0

**Published:** 2015-12-09

**Authors:** N. B. M. M. Rutten, G. T. Rijkers, C. B. Meijssen, C. E. Crijns, J. H. Oudshoorn, C. K. van der Ent, A. M. Vlieger

**Affiliations:** Department of Pediatrics, St Antonius Hospital, PO Box 2500, 3430 EM Nieuwegein, The Netherlands; Department of Sciences, University College Roosevelt Academy, PO Box 94, 4330 AB Middelburg, The Netherlands; Department of Pediatrics, Meander Medical Center, Maatweg 3, 3813 TZ Amersfoort, The Netherlands; Department of Pediatrics, Tergooi Hospital, Rijksstraatweg 1, 1261 AN Blaricum, The Netherlands; Department of Pediatrics, Gelre Hospitals, Albert Schweitzerlaan 31, 7334 DZ Apeldoorn, The Netherlands; Department of Pediatric Pulmonology and Allergology, Wilhelmina Children’s Hospital/University Medical Center, Lundlaan 6, 3584 EA Utrecht, The Netherlands

**Keywords:** Intestinal microbiota, Antibiotics, Infant, Allergic diseases, Microbiota profiling

## Abstract

**Background:**

The acquisition and development of infant gut microbiota can be influenced by numerous factors, of which early antibiotic treatment is an important one. However, studies on the effects of antibiotic treatment in early life on clinical outcomes and establishment and development of the gut microbiota of term infants are limited. Disturbed microbiota composition is hypothesized to be an underlying mechanism of an aberrant development of the immune system. This study aims to investigate the potential clinical and microbial consequences of empiric antibiotic use in early life.

**Methods/Design:**

450 term born infants, of whom 150 are exposed to antibiotic treatment in early life and 300 are not (control group), are included in this observational cohort study with a one-year follow-up. Clinical outcomes, including coughing, wheezing, fever >38 °C, runny nose, glue ear, rash, diarrhea and >3 crying hours a day, are recorded daily by parents and examined by previously defined doctor’s diagnosis. A blood sample is taken at closure to investigate the infant’s vaccination response and sensitization for food and inhalant allergens. Fecal samples are obtained at eight time points during the first year of life. Potential differences in microbial profiles of infants treated with antibiotics versus healthy controls will be determined by use of 16S-23S rRNA gene analysis (IS-pro). Microbiota composition will be described by means of abundance, diversity and (dis)similarity. Diversity is calculated using the Shannon index. Dissimilarities between samples are calculated as the cosine distance between each pair of samples and analyzed with principal coordinate analysis. Clinical variables and possible associations are assessed by appropriate statistics.

**Discussion:**

Both clinical quantitative and qualitative microbial effects of antibiotic treatment in early life may be demonstrated. These findings can be important, since there is evidence that manipulation of the infant microbiota by using pre- or probiotics can restore the ecological balance of the microbiota and may mitigate potential negative effects on the developing immune system, when use of antibiotics cannot be avoided.

**Trial registration:**

ClinicalTrials.gov NCT02536560. Registered 28 August 2015.

## Background

The fetal intestine is (virtually) sterile, however, from birth onwards, the infant intestine becomes colonized with a wide variety of microorganisms [1]. The interaction between the host and its microbiota contributes fundamentally to overall health [2, 3]. For example, the microbial ecosystem provides the host with valuable metabolic features, such as metabolism of otherwise indigestible carbohydrates, xenobiotic metabolism, and production of essential metabolites such as vitamin K [4, 5]. Furthermore, the colonizing microorganisms play a key role in driving post-natal maturation of the infant gut and development of the mucosal immune system [6–10].

Disturbance of the microbial colonization patterns early in life can lead to long-lasting host effects and eventually disease. Aberrancies in microbial colonization patterns or distortion of the microbial ecology early in life might predispose the infant to the development of immune-mediated diseases. The gut microbiota has been associated with T-helper 2 (Th2) type diseases like allergy, wheezing and asthma, and also with T-helper 1 (Th1) type diseases, like inflammatory bowel disease, diabetes and obesity [11–24]. Also in non-immune mediated diseases, like infantile colic or irritable bowel syndrome, the fecal microbiota composition was found to be different from healthy controls [25, 26].

A range of factors can influence the composition of the intestinal microbiota and its establishment, like mode of delivery, feeding mode, contact with parents, siblings, and nursing and/or hospital staff when appropriate [27–29]. Antibiotic treatment during the early postnatal period, that has become common in modern obstetric and neonatal practice [15], is one of the important factors that can influence maturation of the infant gut microbiota and thus increase the risk of disease [11, 24]. A systematic review in 2011 of longitudinal studies on the effects of infant antibiotic use showed a higher risk for subsequent development of wheezing and/or asthma [30]. Other studies have shown an association between infant antibiotic exposure and growth rate and development of adiposity [31–33].

Relatively few studies have determined the direct effects of antibiotics on the composition of gut microbiota and/or addressed the mechanisms underlying this association [13, 29, 34]. Initial studies were based on culture-dependent techniques, but altered intestinal microbiota in antibiotic treated infants could already be identified by these relatively limited techniques [35, 36]. In the last few years, the development of culture-independent (molecular) approaches for studying the intestinal microbiota composition has changed and advanced our original perspective and insight into the impact of the composition of the microbiome [37, 38]. By using molecular fingerprinting and determination of 16S rRNA genes (by quantitative polymerase chain reaction, qPCR), overgrowth of *Enterococci* and arrested growth of *Bifidobacterium* in infants exposed to antibiotics in the first week of life has been described [34].

Overall, evidence is growing that an aberrant microbiota composition as a result of antibiotic treatment can have clinical effects, but more robust studies are needed with a higher number of patients to elucidate the exact effects of antibiotics on the developing microbiota and its association with health in the first year of life. This study therefore aims to investigate the potential clinical and microbial consequences of empiric antibiotic use in early life by following a large cohort of 150 treated and 300 untreated infants and compare them with respect to their health status as well as their developing gut microbiota.

## Methods/Design

### Study design and population

A prospective, observational cohort study with a one-year follow-up is currently conducted to determine the clinical effects and impact on the gut microbiota composition of empiric antibiotic use in early life. Infants are recruited from the maternity wards and neonatal wards of four teaching hospitals in the Netherlands: the St. Antonius Hospital in Nieuwegein, the Tergooi Hospital in Blaricum, the Gelre Hospitals in Apeldoorn and the Meander Medical Center in Amersfoort. This study was approved by the joined Medical Ethics Committee (VCMO) of the St Antonius Hospital and Meander Medical Center (nowadays MEC-U: Medical Research Ethics Committees United (Nieuwegein)). In The Netherlands, since 2013 studies only need to be approved by one Ethics Committee. Subsequently, for other participating centers, proof of local feasibility needs to be given. The approved protocol was consequently checked for local feasibility and expertise at Tergooi Hospital and Gelre Hospitals and permission for study implementation was obtained by the respective boards of directors. The study is performed in accordance with the ethical standards laid down in the 1964 Declaration of Helsinki and its later amendments.

All parents of term infants (≥ 36 weeks of gestational age) who stay in the hospital for at least 24 h, are approached for participation in the study.

In total 150 infants, treated with antibiotics because of (a high suspicion of) a perinatal infection during the first week of life, will be recruited. The control group comprises 300 healthy newborns, born in the hospital and needing clinical observation for 24–48 h for several reasons like maternal comorbidity, low probability of neonatal infection, blood sugar monitoring, meconium containing amniotic fluid, or delivery by caesarean section. For a balanced composition of the control group, as much as possible infants that are suspected to have a neonatal infection but for whom watchful waiting is allowed (and no antibiotic treatment has to be started) will be included.

### Inclusion and exclusion criteria

Inclusion criteria

1. Term-born babies (≥ 36 weeks gestational age)
2. (Short) stay on maternal ward or admission to neonatal ward because of antibiotic treatment
3. Signed informed consent by the parents

Exclusion criteria

1. Congenital illness or malformations
2. Severe perinatal infections for which transfer to the neonatal intensive care unit is needed
3. Maternal probiotic use ≤ six weeks before delivery
4. Insufficient knowledge of the Dutch language.

Infants suspected of neonatal infection will be treated with antibiotics according to the local hospital protocols. All hospitals use gentamycin in combination with either penicillin, amoxicillin or amoxicillin/clavulanic acid. During the study period, type of feeding is closely monitored, as parents monthly report the type of feeding on the calendar list (breast- or formula feeding). Parents are free in their choice of feeding regime, and after six months infants will be on solid food, but not all the infants in the study will have the exact same diet as this is neither feasible nor ethical. Mother’s intake of antibiotics or drugs during pregnancy, delivery and during breastfeeding (if applicable) will be recorded. Delivery/hospital data will be extracted from patient records.

All parents have to give informed consent prior to inclusion in the study. Follow up continues during the first year of life, in which parents collect and store eight fecal samples of their infant. Parents are also asked to collect a fecal sample of their child around the 2^nd^ birthday and they give permission for a possible approach for a follow-up in about 5–6 years.

### Aims

**Fig. 1:**
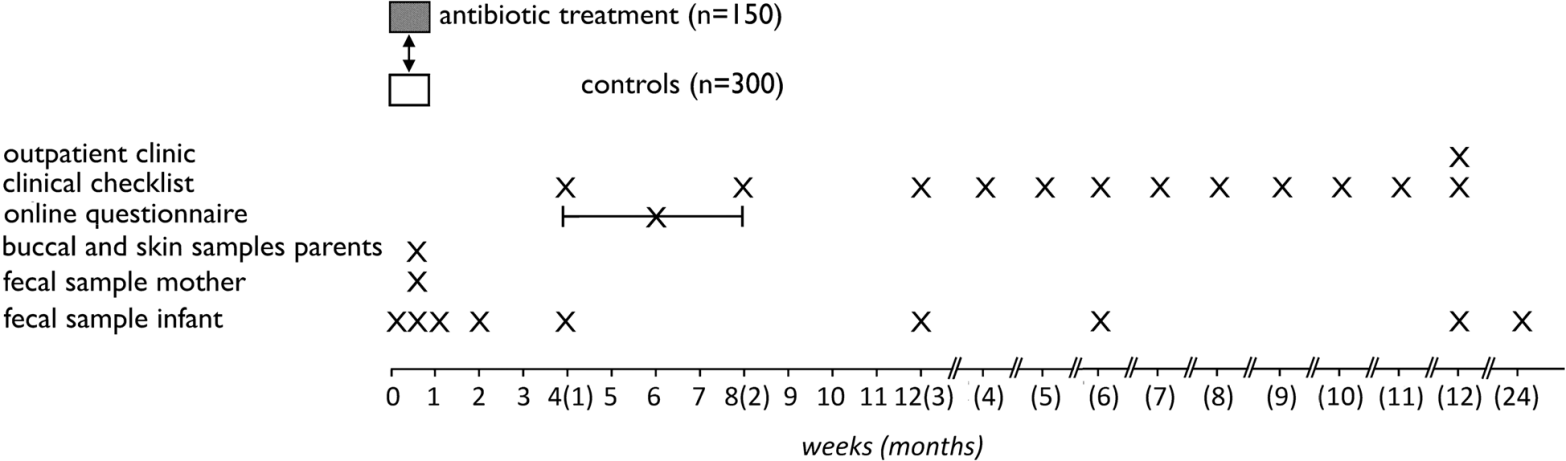
Overview of the sample time points and outcomes

Healthy newborns born in the hospital, observed for low probability of neonatal infection will be compared to newborns exposed to antibiotic therapy in early life (first 1–2 weeks) by investigating potential differences in fecal microbiota composition. For this purpose fecal bacterial composition and diversity is determined at eight time points during the first year of life, from birth on: day one (T1), day two (T2), one week (T3), two weeks (T4), one month (T5), three months (T6), six months (T7), one year (T8). An overview of the sample time points and outcomes is shown in Fig. 1.

Potential differences in proportions (abundance and diversity) of intestinal bacterial groups (phyla) and species in antibiotic treated infants versus healthy controls will be determined by use of 16S-23S rRNA gene analysis. Secondly, we investigate differences in clinical outcomes between infants treated with antibiotics and controls. We evaluate incidences of atopic dermatitis (eczema), food allergy, upper respiratory tract infections (URTI), lower respiratory tract infections (LRTI), gastrointestinal infections (GITI) and excessive crying, prospectively assessed by parental reports and retrospectively assessed by doctor’s diagnoses. The clinical endpoints will be linked to the developing intestinal microbiota during the first year of life. Body mass index and standard deviation (SD)-scores are calculated from individual weight and height curves.

Parents of all included infants will report the use of antibiotics (type of antibiotics and duration of treatment) during the complete study period (i.e. the first year of life) on the calendar lists. Moreover, (the type of) prescribed antibiotics will be investigated at the end of the initial study period by approaching the infant’s pharmacy on medications used in the first year of life. Parents are also asked to report the moment of stool collection (date) on the collection vial, so the investigators can calculate the time between antibiotic treatment and stool sample collection. We hypothesize that in early life antibiotic treated infants develop more eczema during their first year than non-antibiotic treated infants (healthy controls). We also expect an increase in incidence of (parental reported as well as doctor’s diagnosed) food allergy, respiratory tract infections (RTI’s) and gastrointestinal tract infections (GITI’s) in infants treated with antibiotics compared to controls.

### Outcomes

#### Symptoms via calendar checklist

Parents are instructed to record a (for this study) designed daily checklist of clinical symptoms, which include coughing, wheezing, fever >38 °C, runny nose, glue ear, rash, diarrhea, >3 h of crying within one day (24 h). Table 1 shows the various symptoms, which should be recorded by the parents. Descriptions standardize the way of recording as much as possible. The checklists are designed as a calendar and parents are requested to return a list (at the end of) every month. As a motivation for returning the list, we send back a contemporary Dutch magazine for young parents, monthly, after receiving their list.

**Table 1:** Description of symptoms

Coughing	Your infant coughs several times a day and/or has coughing. Regularly there are signs of cold. Don’t record cough because of choking.
Wheezing	During expiration you notice a whistling, wheezy sound coming from the lower airways. During expiration your child is trying to squeeze the air outwards. Don’t record wheezing coming from or through the nose.
Fever >38 °C	Clear from itself, whereby it is important you use a rectal thermometer, measure twice and the temperature is >38 °C on both occasions.
Runny nose	Signs of cold with white/yellow/green mucus running from the nose;
Glue ear	Your child seems to have earache and/or grasps its ear (the ear frequently is high-colored or bends) and/or glue egresses from the ear.
Rash	More than one day existing skin-redness (spots, rash, pimples) on the face, arms or legs, trunk. Disease-symptoms are not necessarily present. The rash can be eczema; eczema mostly is red, moistly, scaly and may itch. Infants cheeks are affected mostly. When children grow up, elbow and knees are preferred sites.
Diarrhea	Increase in stool frequency to twice the usual number per day, that continues more than one day. The content may be watery or mucous.
>3 crying hours a day	Clear from itself, whereby the total crying episodes add up to more than three hours a day (24 h) in total.

At baseline, parents have to fill out an online questionnaire containing questions on demographics, comorbidity and use of medication. This questionnaire includes: gestational age and birthweight of the child, maternal comorbidity (medication during pregnancy and delivery), environmental factors (habitat, housing), parental smoking habits, siblings, pets, chronic diseases and hereditary diseases, ethnicity, education.

Regarding the daily checklists, RTI’s and GITI's are defined as follows: an episode of respiratory tract infection includes at least two consecutive days of coughing and/or wheezing, runny nose and/ or earache (with or without secretion). An episode ends when the child is symptom-free for at least two consecutive days. Diarrhea is defined as increase in stool frequency to twice the usual number per day, during at least two consecutive days. Parents also report visits to the general practitioner because of respiratory or gastrointestinal symptoms of their child (since particularly respiratory infection may have a confounding effect, as antibiotic use and respiratory infections are so common and closely related in early life) [39]. Parents are extensively instructed by one of the investigators, at baseline of the study, how to fill in the daily checklists and how to interpret their child’s symptoms.

#### Symptoms via doctor’s diagnosis

Primary care visits and physicians-diagnoses during the first year of life are recorded according to the International Classification system of Primary Care (ICPC) [40] and are traced through report of the computerized medical files recorded by the general practitioner.

Visits to the physician are defined as the occurrence of a “respiratory ICPC”, “gastrointestinal ICPC” or “dermatological ICPC”. For respiratory ICPC’s, these include dyspnea (R02), wheezing (R03), cough (R05), acute upper tract infection (R74), acute bronchi(oli)tis (R78), pneumonia (R81), asthma like symptoms (R96), or other less prevalent respiratory ICPC’s (breath problems [R04], sneeze [R07], other symptoms of the nose [R08], symptoms of the throat [R21], abnormal sputum [R25], concern about respiratory illness [R27], acute laryngitis [R77], influenza [R88], other infections of the airways [R83], and other respiratory diseases [R99]). For gastrointestinal ICPC’s these include infectious diarrhea (D70), vomiting (D10) and susceptible gastro-intestinal infection (D73). Dermatological ICPC’s include other symptoms/complaints of the skin (S21), dry skin/flaking (S21.01). A child is considered to have eczema when the doctors diagnose was established and (1^st^ or 2^nd^ class) corticosteroids have been prescribed. Prescription will be checked via pharmacist’s medication records. The incidence of colic is recorded as ICPC infantile colic (A14).

#### Outpatient clinic visits

Shortly after the child’s first birthday, all children visit the outpatient clinic of the recruiting hospital and parents are asked to bring the collected, frozen fecal samples (on ice). Disease episodes (as to the respiratory tract and gastrointestinal tract) during the first year of life are discussed and documented. Any prescribed (systemic) antibiotic treatments during the first year of life as well as well as use of probiotics are recorded. At the end of the visit, a venapuncture is performed to collect a 1 ml blood sample. Serum is stored at −20 °C. At the end of the study, IgG antibodies against Tetanus toxoid, Diphtheria toxoid, *Haemophilus influenza* type B, and the capsular polysaccharides of the pneumococcal 10-valent conjugate vaccine will be determined. From the same blood sample, specific IgE to food and inhalant allergens is determined for objective evaluation of allergic sensitization.

#### Collection of fecal samples

Parents collect a total of 8 fecal samples during the first year of life, and one sample at or around the 2^nd^ birthday of the child. Directly after birth, nurses of the maternity and neonatal wards collect the first fecal samples, which are frozen at −20 °C. After discharge the parents start collecting samples at home. Collection of stool samples is standardized (oral instruction by the investigators at time of inclusion and there is a written instruction on the daily checklist of symptoms, that parents receive).

Parents are instructed to sample directly from the diaper by means of stool collection vials (with integrated spoons), and immediately freeze them in their home freezers at a temperature of −18 to −20 °C. Moreover, in order to be able to study transmission of microbiota from the parents to the child, mother collects a stool sample of her own within the first week after the delivery and a buccal mucous membrane swab and a skin swab from the mother and the father are taken at inclusion.

#### 16S-23S IS profiling of the microbiota

The stool samples are subjected to microbiota composition profiling by means of a high-throughput bacterial profiling technique (IS-pro, IS-Diagnostics, Amsterdam, The Netherlands). This technique combines bacterial species differentiation by the length of 16S-23S rRNA interspace region with instant taxonomic classification by phylum-specific fluorescent labeling of PCR-primers. For amplification of IS regions, phylum-specific, fluorescently labeled primers are designed, corresponding to conserved regions within the 16S rRNA and 23S rRNA genes [41]. The procedure consists of two multiplex PCRs, a combination of which provides very broad coverage for *Firmicutes*, *Actinobacteria*, *Fusobacteria* and *Verrucomicrobia* (FAFV), *Bacteroidetes* and *Proteobacteria*. Amplifications are carried out on a GeneAmp PCR system 9700 (Applied Biosystems, Foster City, CA, USA).

Buccal mucous membrane and skin swabs are analyzed by using the same IS-pro technique.

### Data analysis

After pre-processing (IS-pro software suite, IS-Diagnostics), each sample is represented by a microbial profile, consisting of color-labelled peaks. Each peak is characterized by a specific IS fragment (measured as nucleotide length) and a color related to a specific phylum group. The intensity of peaks reflects the relative quantity of PCR product (measured in relative fluorescent units). We further consider each peak as an operational taxonomic unit (OTU) and its corresponding intensity as relative abundance. Intensity values are log2 transformed when appropriate in order to compact the range of variation in peak heights, to reduce the dominance of abundant peaks and include less abundant species of the microbiota in downstream analyses and more related to the in vivo situation. This transformation results in improved consistency of the estimated correlation coefficient, lower impact of inter-run variation, and improved detection of less prominent species. This conversion is used in all downstream analyses, such as calculating within-sample and between-sample microbial diversity [41].

### Statistical analysis

We will evaluate the potential relationships between antibiotic use in early life and the various (clinical) endpoints. Variables are verified for normal distribution and *t*-tests will be used for testing the null hypothesis. Either logistic regression analysis or cox regression analysis will be applied to the data (appearance of the primary endpoint(s) will be recorded as specifically as possible), so that odds ratios or hazard ratios and their 95 % confidence interval can be presented for description of the relationships. Multivariate models will be used to assess confounders and confounding effects. We will perform post hoc analyses to look for potential effects of the different antibiotic regimes. Outcomes may, possibly, be classified into subgroups. According to antibiotic treatment after the first two weeks, children will be grouped based on the number of antibiotic courses during the first year of life (0 / 1 / 2 or more courses). Within the ‘treatment groups’ a distinction can be made between the class or spectrum of the prescribed antibiotics.

Microbiota composition will be described by means of abundance, diversity and (dis)similarity. Diversity is calculated using the Shannon index [42] and differences in this index will be tested with Mann–Whitney *U* test. Dissimilarities between samples, or between-sample diversity, are calculated as the cosine distance between each pair of samples and analyzed with principal coordinate analysis (PCoA). All statistical analysis will be conducted using SPSS version 22.0 (SPSS Inc., Chicago, IL, USA) and the vegan software package in R (Foundation for Statistical Computing, Vienna, Austria).

### Power calculation

The power of this observational cohort study and the number of participants is based on one of the clinical relevant primary endpoints: incidence of eczema after antibiotic exposure in early life. Eczema is the most common inflammatory skin disease of childhood, affecting 5 to 20 % of children at any time. The cumulative prevalence of eczema varies from 20 % in Northern Europe and the USA to 5 % in the south-eastern Mediterranean [43]. In high-risk populations in Northern Europe (e.g. infants with a positive family history of atopy), the incidence of eczema is increased and estimates 60 % [44]. Based on these data, in this study the incidence of eczema in the control group (healthy, non-antibiotic-treated infants) is estimated 20 %, whereas in the antibiotic-treated group (infants we assume at increased risk for developing eczema) the incidence may rise to 35 %. This percentage, to our opinion, reflects a slightly increased risk for developing eczema after antibiotic treatment in early life in a general population of infants. Assuming a dropout rate of 15 %, a total of 450 children (150 antibiotics, 300 controls) have to be included to demonstrate this difference (20 % ↔ 35 %) with a power of 80 % and an *alpha* of 5 %.

The prevalence of other disease outcomes is variable. For example, prevalence rates of infantile colic vary between 5 and 40 % [45], and literature shows that almost half of the children experience wheezing during the first years of life [46]. So far, the effect of antibiotics (prospectively investigated) on these frequencies is unknown. Defining the size of potential differences to capture is therefore limited.

## Discussion

To the best of our knowledge, this is the first prospective observational cohort study, with a follow-up of one year, addressing clinical outcomes after empiric antibiotic treatment in early life which at the same time investigates the development of intestinal microbiota composition after this antibiotic exposure. Subsequently, extensive clinical outcomes can be linked to microbiota composition over time. This study includes a total of 450 infants, of whom 150 are exposed to antibiotic treatment shortly after birth and 300 controls. Previous studies addressing consequences of antibiotic treatment in early life were hampered by a retrospective approach [11, 33, 39, 47], divergent study populations [48], smaller sample sizes [17] or a limited period of follow-up [34, 49].

By assessing an extended number of clinical parameters in a fairly large group, and subsequently strictly selected homogeneous subgroups of infants, bias due to confounding factors such as mode of delivery and type of feeding is expected to be reduced. Results from this study may improve our understanding of the evolving microbiota in term infants. Also, by collecting fecal samples at eight time points during the first year of life and around the second birthday, long-term microbiota development can be investigated.

The fact that children who are sick and receive antibiotic treatment may differ from healthy children, who do not need antibiotic treatment at birth, is one of the most important limitations of this study. However, to our opinion, there are no opportunities to bypass this problem, as ethical concerns prevent us from performing a double blind randomized controlled trial. Another limitation of this study may be the fact that bacterial composition may change as a consequence of freezing fecal samples. For example, levels of *Bacteroidetes* have been shown to be reduced in frozen samples [3, 50, 51]. As all samples will be obtained by the parents and stored in their home freezers, no information on potential differences in storage conditions between samples can be obtained. We try to minimize this variation by instructing (by one of the investigators) all parents on how to collect the faecal samples, which makes the collection procedure as standardized as possible. We do realize that studying the microbiota composition of fecal samples may not be the best method to assess the general composition and the effect of antibiotics in the gastrointestinal tract. Stool samples therefore are used as a proxy for the study of the gut microbiota as these samples are easier to collect than biopsy samples and avoid invasive procedures (and associated ethical issues). A limitation of sampling the gut microbiota is that once the sample is taken, alterations in the relative proportions of various bacterial species can occur so that the sample might no longer accurately reflect the composition of the microbiota in vivo by the time it is processed [3]. This is a well-known problem with respect to molecular approach of microbiota composition and to date it is impossible to collect biopsy samples from infants (and healthy volunteers generally) on a large scale due to the practical and ethical challenges indicated above [3].

IS-pro will be used to characterize the microbiota, because this technique has specifically been designed for application in a clinical setting. It has been validated for clinical diagnostics, which makes it fully reproducible and the high-throughput nature of IS-pro makes analysis of a large number of samples feasible. IS-pro comprises two separate phylum-specific PCR reactions though, which hampers direct comparisons of relative abundances addressing all three phyla together [41]. Next to that, IS-pro does not generate sequence data in the conventional sense. This means IS-pro cannot achieve the same level of detail as next-generation/whole genome sequencing techniques (e.g. Illumina), although sequence data confirmed specificity of IS-pro in (currently) numerous peaks, underlining the validity of the technique [41].

There is now sufficient evidence to conclude that antibiotic treatment in early life has a detrimental effect on the gastrointestinal microbiota composition. However, it is still unclear as to what extent the composition is disturbed and for how long the gastrointestinal dysbiosis remains, if and how microbiota composition will ‘normalize’ and what the effects are on the developing immune system. These findings can be important, since there is evidence that the manipulation of infant microbiota by using pre-or probiotics can restore the ecological balance of the microbiota [34, 52, 53]. If our hypothesis is proven with this study, future studies should demonstrate if targeted intervention, by using dietary supplements, pre- or probiotics or otherwise, may have beneficial effects by limiting the gastrointestinal imbalance due to early antibiotic exposure.

## Abbreviations

GITI: gastrointestinal tract infections
ICPC: International Classification system of Primary Care
LRTI: lower respiratory tract infections
OTU: operational taxonomic unit
PCoA: principal coordinate analysis
PCR: polymerase chain reaction
RTI: respiratory tract infections
SD: standard deviation
URTI: upper respiratory tract infections
